# A rare case of endotracheal tube cuff leakage with no detectable decrease in cuff pressure

**DOI:** 10.1186/s40981-024-00754-9

**Published:** 2024-11-12

**Authors:** Keiichi Nagasawa, Masayuki Nishibata, Sarah Kyuragi Luthe, Tomoyuki Kawamata

**Affiliations:** Department of Anesthesiology, Wakayama Medical University School of Medicine, Wakayama, 641-8510 Japan

**Keywords:** Endotracheal tube, Cuff leak, Pilot balloon tubing, Fiber-optic light, Surgical fire, Ignition source

## Abstract

**Background:**

Common causes of air leakage around an endotracheal tube include insufficient endotracheal tube cuff inflation and damage to the cuff, while damage to the pilot balloon or pilot balloon tubing is relatively rare.

**Case presentation:**

A 74-year-old female with vertebral osteomyelitis was scheduled for an extreme lateral interbody fusion followed posterior fixation. A fiber-optic light was utilized as part of the surgical illuminator. A sudden decrease in tidal volume and airway pressure was noted intraoperatively. We suspected leakage around the endotracheal tube cuff; however, no decrease in cuff pressure was detected. Despite the normal cuff pressure, we decided to inject a small amount of air which led to a significant increase in the cuff pressure. Upon careful inspection of the endotracheal tube, we discovered that the pilot balloon tubing was damaged as a result of thermal energy emitted by the fiber-optic light, which had ignited the surgical drape. The pilot balloon tubing was partially severed in which the section proximal to the endotracheal tube cuff was burned and punctured, causing the cuff leak. Meanwhile, the section proximal to the pilot balloon had melted and occluded the lumen, resulting in a falsely normal cuff pressure reading followed by an elevated cuff pressure when a small amount of air was injected into the pilot balloon during troubleshooting. Appropriate ventilation was resumed after extubation and re-intubation with a new endotracheal tube.

**Conclusions:**

We experienced an endotracheal tube cuff leakage caused by a damaged pilot balloon tubing due to thermal energy of the fiber-optic light. Our case report emphasizes the importance of suspecting damage to the endotracheal tube cuff and inflation system despite a normal cuff pressure reading, given that the measurement may be falsely elevated depending on the specific location of the damage. In addition, all operating personnel should be familiarized with safety warnings and cautions related to handling.

## Background

Air leakage around an endotracheal tube, often described as a cuff leak, is a common complication in mechanically ventilated patients. A cuff leak can lead to inadequate ventilation, resulting in hypercarbia and/or hypoxia [1]. Common causes of a cuff leak with an intact endotracheal tube include underinflation of the endotracheal tube cuff, cephalad migration of the endotracheal tube (partial tracheal extubation), misplaced orogastric or nasogastric tubes, inappropriate tube sizing or discrepancy between the endotracheal tube and trachea, or increased peak airway pressure [2]. Additionally, cuff leaks can also result from damage or manufacturing defects of the endotracheal tube cuff and inflation system including the pilot balloon or pilot balloon tubing [2]. Two previous cases of cuff leaks caused by a damaged to the pilot balloon tubing have been reported [3, 4]. In both cases, cuff leaks were identified by a decrease in cuff pressure measured by a cuff pressure gauge. However, we report a rare case where damage to the pilot balloon tubing resulted in a cuff leak without a detectable decrease in cuff pressure as the section proximal to the endotracheal tube cuff was burned and punctured, while the section proximal to the pilot balloon had melted and occluded the lumen.

## Case presentation

A 74-year-old female (156 cm, 50 kg) with vertebral osteomyelitis was scheduled for an extreme lateral interbody fusion in the lateral decubitus position followed by posterior fixation in the prone positions. In the operating room, standard monitoring including electrocardiography, noninvasive blood pressure, invasive radial artery pressure, percutaneous oxygen saturation, bispectral index, and tympanic temperature was applied. Motor-evoked potential monitoring via transcranial electrical stimulation was planned for the procedure. General anesthesia was induced with propofol and remifentanil. Endotracheal intubation with a cuffed reinforced endotracheal tube with an inner diameter of 7.0 mm was performed following muscle relaxation with rocuronium. The endotracheal tube was secured at a depth of 21 cm on the right side of the mouth with the cuff pressure gauge indicating a cuff pressure of 28 cmH_2_O, and no cuff leak was detected on auscultation. Anesthesia was maintained with propofol, remifentanil, and oxygen-in-air gas mixture. Mechanical ventilation was administered using pressure control ventilation-volume guaranteed with a set tidal volume of 450 mL and a respiratory rate of 10 breaths/min. The patient was placed in the right lateral decubitus position for the extreme lateral interbody fusion. To provide supplemental lighting during surgery, a fiber-optic light attached to a 300-W xenon lamp was utilized as part of the surgical illuminator (ProXenon 350™, Welch Allyn, Inc., New York, USA). The fiber-optic light source intensity was set at 100% (200,000 lx of illumination) throughout the surgery but was stored in a polyethylene electrosurgical safety holster when not in active use. Approximately 1 h into the surgery, we noted a sudden decrease in the tidal volume and airway pressure to less than 50 ml and 5 cmH_2_O, respectively. While ensuring no displacement or obstruction of the endotracheal tube, an intact respiratory circuit, and no malfunction of equipment failure, we suspected air leakage around the endotracheal tube cuff; however, no decrease was detected.

**Fig. 1:**
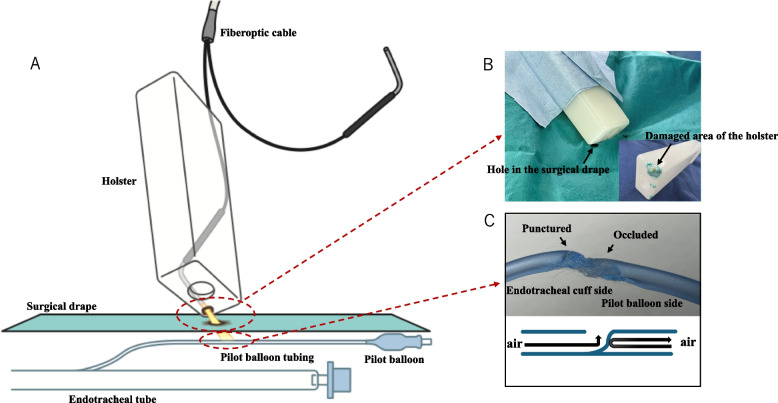
Thermal damage induced by the fiber-optic light. **A** A diagram illustrating thermal damage caused by the fiber-optic light. The thermal energy emitted from the illuminated end of the fiber-optic light burned the bottom of the electrosurgical safety holster in which the fiber-optic light was stored, subsequently igniting the surgical drape beneath the holster and leading to damage of the pilot balloon tubing. **B** The damaged holster and surgical drape caused by thermal damage from the fiber-optic light. An inset image highlights the damaged section of the electrosurgical safety holster. The arrowhead and arrow indicate the holes in the surgical drape and the holster, respectively. **C** The damaged pilot balloon tubing caused by thermal damage from the fiber-optic light. The upper panel shows the damaged pilot balloon tubing, and the lower panel provides a schematic representation of the damage. The pilot balloon tubing was partially severed in which the section proximal to the endotracheal tube cuff was burned and punctured, causing the cuff leak. Meanwhile, the section proximal to the pilot balloon had melted and occluded the lumen, resulting in a falsely normal cuff pressure reading followed by an elevated cuff pressure when a small amount of air was injected into the pilot balloon during troubleshooting

The cuff pressure gauge was maintained at 28 cmH_2_O, consistent with the level immediately after intubation. Despite the normal cuff pressure, we decided to inject a small amount of air which led to a significant increase in the cuff pressure. Upon careful inspection of the endotracheal tube, we discovered that the pilot balloon tubing was damaged as a result of thermal energy emitted by the illuminated end of the fiber-optic light (Fig. 1A). This energy burned the bottom of the electrosurgical safety holster in which the fiberoptic light was stored, subsequently igniting the surgical drape beneath the holster (Fig. 1A, B) and leading to damaging the pilot balloon tubing. More specifically, the pilot balloon tubing was partially severed in which the section proximal to the endotracheal tube cuff was burned and punctured, causing the cuff leak (Fig. 1C). Meanwhile, the section proximal to the pilot balloon had melted and occluded the lumen, resulting in a falsely normal cuff pressure reading followed by an elevated cuff pressure when a small amount of air was injected into the pilot balloon during troubleshooting (Fig. 1C).

Appropriate ventilation was resumed after extubation and re-intubation with a new endotracheal tube. Subsequently, the surgery was successfully completed, with no burns observed on the patient’s body. The postoperative course was uneventful, and the patient was discharged on postoperative day 21. This case was reviewed by the Medical Safety Promotion Department of our institution, and relevant departments were notified.

## Discussion

Electrosurgical devices including electrocautery, various lasers, and fiber-optic light sources are well-known ignition sources leading to surgical fires and thermal injuries in the operating room. One previous study examined the response of polypropylene drapes to fiber-optic light cables, indicating burn risk when the cable was buried within the drape regardless of oxygen sources [5]. However, to the best of our knowledge, this is the first case reporting thermal damage to endotracheal tube caused by a fiber-optic light source.

Additionally, this case is unique given that the pilot balloon tubing was partially severed and punctured on the endotracheal tube cuff side but occluded on the pilot balloon side, leading to a cuff leak without a decrease in cuff pressure reading, complicating the troubleshooting process for the decreased tidal volume and peak airway pressure. The falsely elevated cuff pressure reading after a small amount of air injected into the pilot balloon is consistent with these findings.

Moreover, recent literature indicates that the temperature at the tip of the fiber-optic light can rise from 80 to 160 °C when the intensity is increased from 25 to 50% using a 300-W xenon lamp as the light source, with the temperature reaching up to 246 °C at 100% intensity [6]. Further, a review of the instructions for the specific surgical illuminator used in our case with the fiber-optic light revealed safety warnings and cautions about the risk of fire ignition when energized fiber-optic cables were placed on flammable materials [7]. Furthermore, the manufacturer recommends turning off the device when not in active use, avoiding storage in the holster during surgery, and setting the intensity to 50% while the device is actively in use. Therefore, we believe this case presents an educational opportunity for all operating personnel to become familiar with the safety warnings and cautions related to surgical equipment.

In conclusion, we report a case of endotracheal tube cuff leakage caused by damage to the pilot balloon tubing due to thermal energy of the fiber-optic light. Our case report emphasizes the importance of suspecting damage to the endotracheal tube cuff and/or inflation system despite a normal cuff pressure reading, given that the measurements may be falsely elevated depending on the specific location of the damage.

## Data Availability

Not applicable.
